# Adaptive Fixed-Time Neural Network Tracking Control of Nonlinear Interconnected Systems

**DOI:** 10.3390/e23091152

**Published:** 2021-09-01

**Authors:** Yang Li, Jianhua Zhang, Xinli Xu, Cheng Siong Chin

**Affiliations:** 1 School of Information and Control Engineering, Qingdao University of Technology, Qingdao 266525, China; yang_li@qut.edu.cn (Y.L.); xuxinli@qut.edu.cn (X.X.); 2 Faculty of Science, Agriculture, and Engineering, Newcastle University Singapore, Singapore 599493, Singapore; cheng.chin@ncl.ac.uk

**Keywords:** adaptive fixed-time, neural network, nonlinear interconnected systems

## Abstract

In this article, a novel adaptive fixed-time neural network tracking control scheme for nonlinear interconnected systems is proposed. An adaptive backstepping technique is used to address unknown system uncertainties in the fixed-time settings. Neural networks are used to identify the unknown uncertainties. The study shows that, under the proposed control scheme, each state in the system can converge into small regions near zero with fixed-time convergence time via Lyapunov stability analysis. Finally, the simulation example is presented to demonstrate the effectiveness of the proposed approach. A step-by-step procedure for engineers in industry process applications is proposed.

## 1. Introduction

In actual industrial processes, after decades of development, several control strategies based on classical control theory and modern control theory have been developed. However, most of these control methods are based on single-input single-output linear systems. There are many nonlinear, uncertain, unmodeled dynamic problems in actual industrial processes that pose great challenges to the design of control systems. With the development of engineering automation requirements, the research of control strategies based on multi-input multi-output nonlinear systems has attracted growing attention. In recent decades, many control schemes have been proposed for stability analysis and the control for nonlinear systems, such as the adaptive technique [1,2,3], backstepping technique [4,5,6], U model control [7,8,9], sliding mode control [10,11,12], super twisting algorithm [13,14], neural network technique [6,15,16], etc. In particular, neural network technology has attracted many researchers’ attention because of the following aspects: (1) a neural network has the strong ability to learn any function and can approximate any nonlinear system, and (2) because of the self-learning ability of neural networks, the controller does not need much system model and parameter information, so neural network control can be widely used to solve the control problems caused by uncertain models [17]. In [18], the control problem of time-varying output constraints was investigated using the neural network technique. The use of adaptive neural network control for an uncertain nonlinear system with external disturbance was presented in [19]. In [20], neural network controller designs were presented for several classes of nonlinear systems, including single-input single-output nonlinear systems, strict feedback nonlinear systems, nonaffine nonlinear systems, and multi-input multi-output triangular nonlinear systems.

Most of the above research was proven based on Lyapunov stability theory. However, actual systems often have various disturbances that cannot strictly meet the definition of Lyapunov stability. To solve this problem, scholars have mainly introduced new concepts based on two aspects. On the one hand, the concept of input-to-state stability has been introduced; on the other hand, the concept of practical stability has been introduced with the aim of making systems stable in finite time. If a closed-loop system reaches a stable state in a limited time, it is called finite-time stability. Further, if a system meets the convergence time and does not depend on the initial parameters, it is called fixed-time stability. Compared with the traditional finite-time control method, the convergence time of the fixed-time control method is independent of the initial conditions. Exponential stability, finite-time stability, and fixed-time stability are all concepts related to the convergence rate of a system. They are very important for many control applications, such as the explosion of missiles. In [21], taking the stability analysis of a sliding mode control system as an example, input–output stability, finite-time stability, and fixed-time stability are introduced in detail. In [14], the convergence time of the super-twisting algorithm for a nonaffine nonlinear system was calculated. In [22], the finite time input–output stability of nonlinear systems is studied. In [23], a finite-time adaptive controller is designed for interconnected systems with time-varying output constraints to make the system stable in finite time, but the fixed-time convergence problem is not considered in this paper. The use of adaptive fixed-time tracking control for a strict feedback nonlinear system was studied in [24]. However, there are still many problems to be solved in these existing control strategies, such as state constraints, the adaptive backstepping “explosion” problem, and so on.

In the actual production process, many physical models, such as power systems, process control systems, and manipulator models, can be modeled as nonlinear interconnected systems. Because the interconnected terms between nonlinear interconnected subsystems are unknown, it is physically difficult to obtain this information through sensors, and it requires significant computer resources. Therefore, designing a reliable control scheme for nonlinear interconnected systems is a challenging task. The adaptive decentralized control scheme for interconnected nonlinear systems was discussed in [25]. In [26], the robust adaptive tracking control scheme was proposed for uncertain interconnected nonlinear systems. In [27], the use of adaptive neural control for high-order interconnected systems was examined.

Based on the above analysis, the main goal of this article is to design an adaptive fixed-time neural network tracking controller for nonlinear interconnected systems. The main contributions of this paper are as follows:

(1) The combination of fixed-time control and neural network adaptive control for nonlinear interconnected systems.
(2) A fixed-time low pass filter is designed to solve the “explosion of complexity” based on backstepping control technology.
(3) A fixed-time controller is designed, which contains the convergence time of the error system, weights of neural networks, and a low pass filter system.

The article is organized into the following sections. A nonlinear interconnected mathematical description of the problem is presented in Section 2, the adaptive fixed-time neural network control scheme for a class of nonlinear interconnected systems is proposed in Section 3, two simulation examples are provided to show the reliability of the presented control scheme in Section 4, and finally, some conclusions are given in Section 5.

## 2. Problem Formation and Preliminaries

Consider the interconnected nonlinear system:

(1) $$\begin{array}{l} {\dot{x}}_{i,m}=x_{i,m+1}+f_{i,m}\left({\bar{x}}_{i,m}\right)\\ {\dot{x}}_{i,n}=u_{i}+f_{i,n}\left({\bar{x}}_{i,n}\right)+h_{i,n}\left({\bar{x}}_{1,n},{\bar{x}}_{2,n},\cdots ,{\bar{x}}_{N,n}\right)\\ y_{i}=x_{i,1} \end{array}$$

where $x_{i,m}\in R$ is the state of the interconnected nonlinear system; ${\bar{x}}_{i,m}={\left[x_{i,1},\ldots ,x_{i,m}\right]}^{T}\in R^{m}$ is the state vector of the system; $f_{i,m}\left({\bar{x}}_{i,m}\right):R^{m}\to R$ is the known smooth function; $h_{i,n}\left({\bar{x}}_{1,n},{\bar{x}}_{2,n},\cdots ,{\bar{x}}_{N,n}\right):R^{n\times N}\to R$ is the unknown smooth function; $y_{i}\in R$ is the output of the system; $u_{i}\in R$ is the corresponding control input of the system; the desired trajectory $y_{i,d}$ and its derivative are continuous and bounded.

**Remark** **1:** *In the next section, we introduce a neural network adaptive control method based on the fixed-time stability theory. The objective of the method is for the nonlinear interconnected system output to be able to track the desired signal and maintain fixed-time stability based on the adaptive fixed-time neural network controller. The designed setting time does not rely on the initial parameters and can be realized only by adjusting the controller parameters.*

## 3. Adaptive Fixed-Time Tracking Control System Design

### 3.1. Control System Design

In this section, the design of a fixed-time adaptive law for the error systems of neural networks will be presented. The tracking control system’s objective is to drive the error system to fixed-time stability. To solve the tracking control problem, the neural network adaptive controller, based on fixed-time Lyapunov stability theory for nonlinear interconnected systems, is presented. The adaptive fixed-time laws were designed to update the weights of the neural networks for the error systems. Neural networks were used to approximate unknown functions. The parameters of the neural networks were iteratively based on the Lyapunov fixed-time stability theorem. The convergence time can be designed by choosing controller parameters without the initial condition. Based on the controller, the error-closed loop system achieves Lyapunov fixed-time bounded stability, which means the output trajectory can track to the desired trajectory in fixed time.

**Figure 1:**
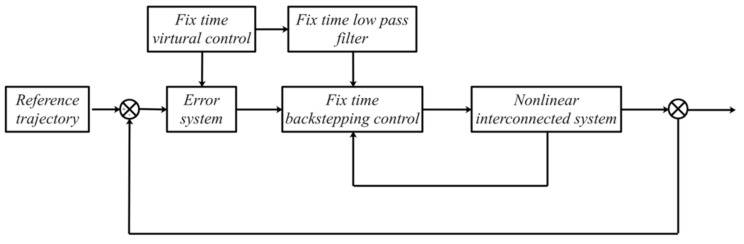
Control structure of the closed system.

**Remark** **2:** *The control structure’s design for the closed loop system is shown in Figure 1. The states of the error system can be determined from the minus between the setting reference function and the actual output function of the nonlinear interconnected system.*

### 3.2. Control System Analysis

Step 1: First, for the system *i*, the following variables are selected:

(2) $$z_{i,1}=x_{i,1}-y_{i,d}$$

The dynamics of $z_{i,1}$ can be obtained as

(3) $${\dot{z}}_{i,1}=x_{i,2}+f_{i,1}\left(x_{i,1}\right)-{\dot{y}}_{i,d}$$

Design the ideal virtual control as

(4) $${\bar{\alpha }}_{i,1}=-p_{i,1}z_{i,1}^{p}-q_{i,1}z_{i,1}^{q}-f_{i,1}\left(x_{i,1}\right)+{\dot{y}}_{i,d}$$

where $p_{i,1}>0,q_{i,1}>0$ Select the virtual control $\alpha_{i,1}$ and design the adaptive law as

(5) $${\dot{\alpha }}_{i,1}=-l_{i,p,1}y_{i,1}^{p}-l_{i,q,1}y_{i,1}^{q}-k_{i,\alpha ,1}y_{i,1}-z_{i,1},\alpha_{i,1}\left(0\right)=0$$

where, $l_{i,p,1}>0,l_{i,q,1}>0$ and the error virtual control is

(6) $$y_{i,1}=\alpha_{i,1}-{\bar{\alpha }}_{i,1}$$

Therefore, based on system (3) and virtual control

(7) $$\begin{array}{cc} {\dot{z}}_{i,1} & =x_{i,2}+f_{i,1}\left(x_{i,1}\right)-{\dot{y}}_{i,d}-p_{i,1}z_{i,1}^{p}-q_{i,1}z_{i,1}^{q}-f_{i,1}\left(x_{i,1}\right)+{\dot{y}}_{i,d}-{\bar{\alpha }}_{i,1}\\ & =x_{i,2}-p_{i,1}z_{i,1}^{p}-q_{i,1}z_{i,1}^{q}-\alpha_{i,1}+\alpha_{i,1}-{\bar{\alpha }}_{i,1}\\ & =-p_{i,1}z_{i,1}^{p}-q_{i,1}z_{i,1}^{q}+z_{i,2}+y_{i,1} \end{array}$$

where

(8) $$z_{i,2}=x_{i,2}-\alpha_{i,1}$$

and the dynamic error virtual control is

(9) $$\begin{array}{cc} {\dot{y}}_{i,1} & ={\dot{\alpha }}_{i,1}-{\dot{\bar{\alpha }}}_{i,1}\\ & =-l_{i,p,1}y_{i,1}^{p}-l_{i,q,1}y_{i,1}^{q}-k_{i,\alpha ,1}y_{i,1}-z_{i,1}-{\dot{\bar{\alpha }}}_{i,1} \end{array}$$

The Lyapunov candidate functional is chosen as

(10) $$V_{1}=\frac{1}{2}z_{i,1}^{2}+\frac{1}{2}y_{i,1}^{2}$$

Differentiating $V_{1}$ with respect to time t yields

(11) $$\begin{array}{cc} {\dot{V}}_{1} & =z_{i,1}{\dot{z}}_{i,1}+y_{i,1}{\dot{y}}_{i,1}\\ & =-p_{i,1}z_{i,1}^{p+1}-q_{i,1}z_{i,1}^{q+1}+z_{i,1}z_{i,2}\\ & -l_{i,p,1}y_{i,1}^{p+1}-l_{i,q,1}y_{i,1}^{q+1}-k_{i,\alpha ,1}y_{i,1}^{2}-y_{i,1}{\dot{\bar{\alpha }}}_{i,1} \end{array}$$

Step *m*: the form of the tracking error is

(12) $$z_{i,m}=x_{i,m}-\alpha_{i,m-1}$$

The dynamics of $z_{i,m}$ can be obtained as

(13) $${\dot{z}}_{i,m}=x_{i,m+1}+f_{i,m}\left(x_{i,m}\right)-{\dot{\alpha }}_{i,m-1}$$

Design the ideal virtual control as

(14) $${\bar{\alpha }}_{i,m}=-p_{i,m}z_{i,m}^{p}-q_{i,m}z_{i,m}^{q}-f_{i,m}\left(x_{i,m}\right)+{\dot{\alpha }}_{i,m-1}$$

where $p_{i,m}>0,q_{i,m}>0$ Select the virtual control $\alpha_{i,m}$, and the adaptive law can be obtained as

(15) $${\dot{\alpha }}_{i,m}=-l_{i,p,m}y_{i,m}^{p}-l_{i,q,m}y_{i,m}^{q}-k_{i,\alpha ,m}y_{i,m}-z_{i,m},\alpha_{i,m}\left(0\right)=0$$

where, $l_{i,p,m}>0,l_{i,q,m}>0$ and the error virtual control is

(16) $$y_{i,m}=\alpha_{i,m}-{\bar{\alpha }}_{i,m}$$

Therefore, based on system (13) and virtual control

(17) $$\begin{array}{cc} {\dot{z}}_{i,m} & =x_{i,m+1}+f_{i,m}\left(x_{i,m}\right)-{\dot{\alpha }}_{i,m-1}-p_{i,m}z_{i,m}^{p}-q_{i,m}z_{i,m}^{q}-f_{i,m}\left(x_{i,m}\right)+{\dot{y}}_{i,m-1}-{\dot{\alpha }}_{i,m-1}\\ & =x_{i,m+1}-p_{i,m}z_{i,m}^{p}-q_{i,m}z_{i,m}^{q}-\alpha_{i,m}+\alpha_{i,m}-{\bar{\alpha }}_{i,m}\\ & =-p_{i,m}z_{i,m}^{p}-q_{i,m}z_{i,m}^{q}+z_{i,m+1}+y_{i,m} \end{array}$$

where

(18) $$z_{i,m+1}=x_{i,m+1}-\alpha_{i,m}$$

and the dynamic error virtual control is

(19) $$\begin{array}{cc} {\dot{y}}_{i,m} & ={\dot{\alpha }}_{i,m}-{\dot{\bar{\alpha }}}_{i,m}\\ & =-l_{i,p,m}y_{i,m}^{p}-l_{i,q,m}y_{i,m}^{q}-k_{i,\alpha ,m}y_{i,m}-z_{i,m}-{\dot{\bar{\alpha }}}_{i,m} \end{array}$$

The Lyapunov candidate functional is chosen as

(20) $$V_{m}=\frac{1}{2}z_{i,m}^{2}+\frac{1}{2}y_{i,m}^{2}$$

Differentiating $V_{m}$ with respect to time t yields

(21) $$\begin{array}{cc} {\dot{V}}_{m} & =z_{i,m}{\dot{z}}_{i,m}+y_{i,m}{\dot{y}}_{i,m}\\ & =-z_{i,m-1}z_{i,m}-p_{i,m}z_{i,m}^{p+1}-q_{i,m}z_{i,m}^{q+1}+z_{i,m}z_{i,m+1}\\ & -l_{i,p,m}y_{i,m}^{p+1}-l_{i,q,m}y_{i,m}^{q+1}-k_{i,\alpha ,m}y_{i,m}^{2}-y_{i,m}{\dot{\bar{\alpha }}}_{i,m} \end{array}$$

Step *n*: the time derivative of $z_{i,n}$ can be described as

(22) $$z_{i,n}=x_{i,n}-\alpha_{i,n-1}$$

Based on dynamics and tracking errors, the dynamics of $z_{i,n}$ can be obtained as

(23) $${\dot{z}}_{i,n}=u_{i}+f_{i,n}\left({\bar{x}}_{i,n}\right)+h_{i,n}\left({\bar{x}}_{1,n},{\bar{x}}_{2,n},\cdots ,{\bar{x}}_{N,n},\right)-{\dot{\alpha }}_{i,n-1}$$

Design the NNs’ approximate nonlinear systems as

(24) $$h_{i,n}\left({\bar{x}}_{1,n},{\bar{x}}_{2,n},\cdots ,{\bar{x}}_{N,n}\right)=W_{i}^{T}\Psi \left(Z\right)+\varepsilon_{i}\left(Z\right)$$

where $Z={\left[{\bar{x}}_{1,n},{\bar{x}}_{2,n},\cdots ,{\bar{x}}_{N,n}\right]}^{T}$, $\theta_{i}=\left\|W_{i}\right\|$, and is estimated by ${\hat{\theta }}_{i}$. The controller is designed as

(25) $$u_{i}=-z_{i,n-1}-p_{i,n}z_{i,n}^{p}-q_{i,n}z_{i,n}^{q}-k_{i,n}z_{i,n}-f_{i,n}\left({\bar{x}}_{i,n}\right)-sign\left(z_{i,n}\right){\hat{\theta }}_{i}\left\|\Psi \right\|+{\dot{\alpha }}_{i,n-1}$$

where $k_{i,n}>\frac{1}{2}$. Design the adaptive law as

(26) $${\dot{\hat{\theta }}}_{i}=\mu_{i}\left(\left|z_{i,n}\right|\left|\Psi_{i}\right|-\varsigma_{i}{\hat{\theta }}_{i}^{p}-\chi_{i}{\hat{\theta }}_{i}^{q}\right)$$

where $\mu_{i}>0,\varsigma_{i}>0,\chi_{i}>0$ Therefore, based on system (23) and controller

(27) $${\dot{z}}_{i,n}=W_{i}^{T}\Psi \left(Z\right)+\varepsilon_{i}\left(Z\right)-z_{i,n-1}-p_{i,n}z_{i,n}^{p}-q_{i,n}z_{i,n}^{q}-k_{i,n}z_{i,n}-sign\left(z_{i,n}\right){\hat{\theta }}_{i}\left\|\Psi \right\|$$

the Lyapunov candidate functional is chosen as

(28) $$V_{n}=\frac{1}{2}z_{i,n}^{2}+\frac{1}{2\mu_{i}}{\tilde{\theta }}_{i}^{2}$$

Differentiating $V_{n}$ with respect to time t yields

(29) $$\begin{array}{cc} {\dot{V}}_{n} & =z_{i,n}{\dot{z}}_{i,n}+\frac{1}{\mu_{i}}{\tilde{\theta }}_{i}{\dot{\hat{\theta }}}_{i}\\ & =z_{i,n}W_{i}^{T}\Psi \left(Z\right)+z_{i,n}\varepsilon_{i}\left(Z\right)-z_{i,n-1}z_{i,n}-p_{i,n}z_{i,n}^{p+1}-q_{i,n}z_{i,n}^{q+1}\\ & -k_{i,n}z_{i,n}^{2}-{\hat{\theta }}_{i}\left|z_{i,n}\right|∥\Psi ∥+{\tilde{\theta }}_{i}\left(\left|z_{i,n}\right|\left|\Psi_{i}\right|-\varsigma_{i}{\hat{\theta }}_{i}^{p}-\chi_{i}{\hat{\theta }}_{i}^{q}\right)\\ & \leq \theta_{i}\left|z_{i,n}\right|∥\Psi ∥-\left(k_{i,n}-\frac{1}{2}\right)z_{i,n}^{2}+\frac{1}{2}{\bar{\varepsilon }}_{i}-z_{i,n-1}z_{i,n}-p_{i,n}z_{i,n}^{p+1}-q_{i,n}z_{i,n}^{q+1}\\ & -{\hat{\theta }}_{i}\left|z_{i,n}\right|∥\Psi ∥+{\tilde{\theta }}_{i}\left|z_{i,n}\right|\left|\Psi_{i}\right|-\varsigma_{i}{\tilde{\theta }}_{i}{\hat{\theta }}_{i}^{p}-\chi_{i}{\tilde{\theta }}_{i}{\hat{\theta }}_{i}^{q} \end{array}$$

where $\varepsilon_{i}^{2}\left(Z\right)\leq {\bar{\varepsilon }}_{i}$. Based on Lemma 4, we have

(30) $$\begin{array}{l} -\varsigma_{i}{\tilde{\theta }}_{i}{\hat{\theta }}_{i}^{p}\leq -\gamma_{i}{\tilde{\theta }}_{i}^{p+1}+\lambda_{i}\theta_{i}^{p+1}\\ -\chi_{i}{\tilde{\theta }}_{i}{\hat{\theta }}_{i}^{q}\leq -\nu_{i}{\tilde{\theta }}_{i}^{q+1}+\upsilon_{i}\theta_{i}^{q+1} \end{array}$$

where $\varsigma_{i},\gamma_{i},\lambda_{i},\chi_{i},\nu_{i},\upsilon_{i}$ are real numbers, $\gamma_{i},\lambda_{i}$ is determined by $\varsigma_{i},p$, and $\nu_{i},\upsilon_{i}$ is determined by $\chi_{i},q$. Therefore, we have

(31) $$\begin{array}{cc} {\dot{V}}_{n}\leq & -\left(k_{i,n}-\frac{1}{2}\right)z_{i,n}^{2}+\frac{1}{2}{\bar{\varepsilon }}_{i}-z_{i,n-1}z_{i,n}-p_{i,n}z_{i,n}^{p+1}-q_{i,n}z_{i,n}^{q+1}\\ & -\gamma_{i}{\tilde{\theta }}_{i}^{p+1}+\lambda_{i}\theta_{i}^{p+1}-\nu_{i}{\tilde{\theta }}_{i}^{q+1}+\upsilon_{i}\theta_{i}^{q+1} \end{array}$$

**Theorem** **1.** *For the interconnected nonlinear system (1), based on the feasible virtual control signal (5), (15) actual controller (25), and adaptive law (26), the error state between the system output and the desired function is fixed-time Lyapunov stability, and the setting time does not rely on the initial parameters.*

**Proof.**  Based on the Lyapunov candidate functionals (10), (20), and (28), differentiating the Lyapunov functional with respect to time t yields (11), (21), and (31). Choosing the Lyapunov candidate functional as

(32) $$V=\frac{1}{2}\sum_{j=1}^{n}z_{i,j}^{2}+\frac{1}{2}\sum_{j=1}^{n-1}y_{i,j}^{2}+\frac{1}{2\mu_{i}}{\tilde{\theta }}_{i}^{2}$$

and differentiating the Lyapunov functional with respect to time t yields

(33) $$\begin{array}{cc} \dot{V}\leq & -\sum_{j=1}^{n}p_{i,j}z_{i,j}^{p+1}-\sum_{j=1}^{n}q_{i,j}z_{i,j}^{q+1}-\sum_{j=1}^{n}l_{i,p,j}y_{i,j}^{p+1}-\sum_{j=1}^{n}l_{i,q,j}y_{i,j}^{q+1}\\ & -\sum_{j=1}^{n-1}k_{i,\alpha ,j}y_{i,j}^{2}-\sum_{j=1}^{n-1}y_{i,j}{\dot{\bar{\alpha }}}_{i,j}-\left(k_{i,n}-\frac{1}{2}\right)z_{i,n}^{2}+\frac{1}{2}{\bar{\varepsilon }}_{i}\\ & -\gamma_{i}{\tilde{\theta }}_{i}^{p+1}+\lambda_{i}\theta_{i}^{p+1}-\nu_{i}{\tilde{\theta }}_{i}^{q+1}+\upsilon_{i}\theta_{i}^{q+1} \end{array}$$

where ${\left|{\dot{\bar{\alpha }}}_{i,1}\right|}^{2}\leq p_{i}$, and

(34) $$\begin{array}{cc} \dot{V}\leq & -\sum_{j=1}^{n}p_{i,j}z_{i,j}^{p+1}-\sum_{j=1}^{n}q_{i,j}z_{i,j}^{q+1}-\sum_{j=1}^{n}l_{i,p,j}y_{i,j}^{p+1}-\sum_{j=1}^{n}l_{i,q,j}y_{i,j}^{q+1}\\ & -\gamma_{i}{\tilde{\theta }}_{i}^{p+1}-\nu_{i}{\tilde{\theta }}_{i}^{q+1}-\sum_{j=1}^{n-1}\left(k_{i,\alpha ,j}-\frac{1}{2}\right)y_{i,j}^{2}-\left(k_{i,n}-\frac{1}{2}\right)z_{i,n}^{2}\\ & +\sum_{j=1}^{n-1}m_{j}+\frac{1}{2}{\bar{\varepsilon }}_{i}+\lambda_{i}\theta_{i}^{p+1}+\upsilon_{i}\theta_{i}^{q+1} \end{array}$$

By choosing the control parameters, $k_{i,\alpha ,j}>\frac{1}{2},k_{i,n}>\frac{1}{2}$ we have

(35) $$\begin{array}{cc} \dot{V}\leq & -\sum_{j=1}^{n}p_{i,j}z_{i,j}^{p+1}-\sum_{j=1}^{n}q_{i,j}z_{i,j}^{q+1}-\sum_{j=1}^{n}l_{i,p,j}y_{i,j}^{p+1}\\ & -\sum_{j=1}^{n}l_{i,q,j}y_{i,j}^{q+1}-\gamma_{i}{\tilde{\theta }}_{i}^{p+1}-\nu_{i}{\tilde{\theta }}_{i}^{q+1}\\ & +\sum_{j=1}^{n-1}m_{j}+\frac{1}{2}{\bar{\varepsilon }}_{i}+\lambda_{i}\theta_{i}^{p+1}+\upsilon_{i}\theta_{i}^{q+1}\\ & \leq -aV^{\frac{p+1}{2}}-bV^{\frac{q+1}{2}}+c \end{array}$$

where

(36) $$\begin{array}{c} a=\frac{\min\left\{p_{i,j\in N},l_{i,p,j\in N},\gamma_{i}\right\}}{{\left(\max\left\{\frac{1}{2},\frac{1}{2\mu_{i}}\right\}\right)}^{\frac{p+1}{2}}},b=\frac{{\left(2n\right)}^{\frac{1-q}{2}}\min\left\{q_{i,j\in N},l_{i,q,j\in N},\nu_{i}\right\}}{{\left(\max\left\{\frac{1}{2},\frac{1}{2\mu_{i}}\right\}\right)}^{\frac{q+1}{2}}}\\ c=\sum_{j=1}^{n-1}m_{j}+\frac{1}{2}{\bar{\varepsilon }}_{i}+\lambda_{i}\theta_{i}^{p+1}+\upsilon_{i}\theta_{i}^{q+1} \end{array}$$

Therefore, the error system is fixed-time, practical, and stable. □

**Remark** **3:** *From the definition of Lyapunov stability, Lyapunov stability, asymptotic stability, and finite-time stability are the three most basic concepts, and their definitions are progressive.*

*At present, the concepts of stability related to the convergence rate mainly include exponential stability, finite-time stability, and fixed-time stability. Exponential stability is the realization of asymptotic stability, while fixed-time stability is the generalization of finite-time stability. From the point of view of standard definitions, they are strictly distinguished, and from the point of view of controller design, they also have different forms.*

*From the perspective of system convergence time, Lyapunov stability can be divided into infinite-time stability and finite-time stability. In the theoretical analysis of stability, the most common exponential stability is infinite-time stability. Infinite-time stability means that when time tends to infinity, the system state can converge exponentially by designing the parameters of the controller. Finite-time stability means that the system can be stable at a certain time and continue to maintain a stable state by designing the parameters of the controller. For practical engineering control, finite-time stability is obviously more practical than infinite-time stability, but there are some limitations to finite-time stability; for instance, the convergence time of the designed system depends on the initial state. Therefore, in this section, another scheme was introduced: the tracking control method for nonlinear interconnected systems based on fixed-time stability theory. The setting time does not depend on the initial parameters and can be realized only by adjusting the controller parameters. In other words, under the condition that the controlled system is stable in fixed time, even if the initial parameters are changed, the controlled system can still be stable within the originally designed fixed time without redesigning the controller. This greatly improves the potential of the control method to be used in practical applications.*

**Figure 2:**
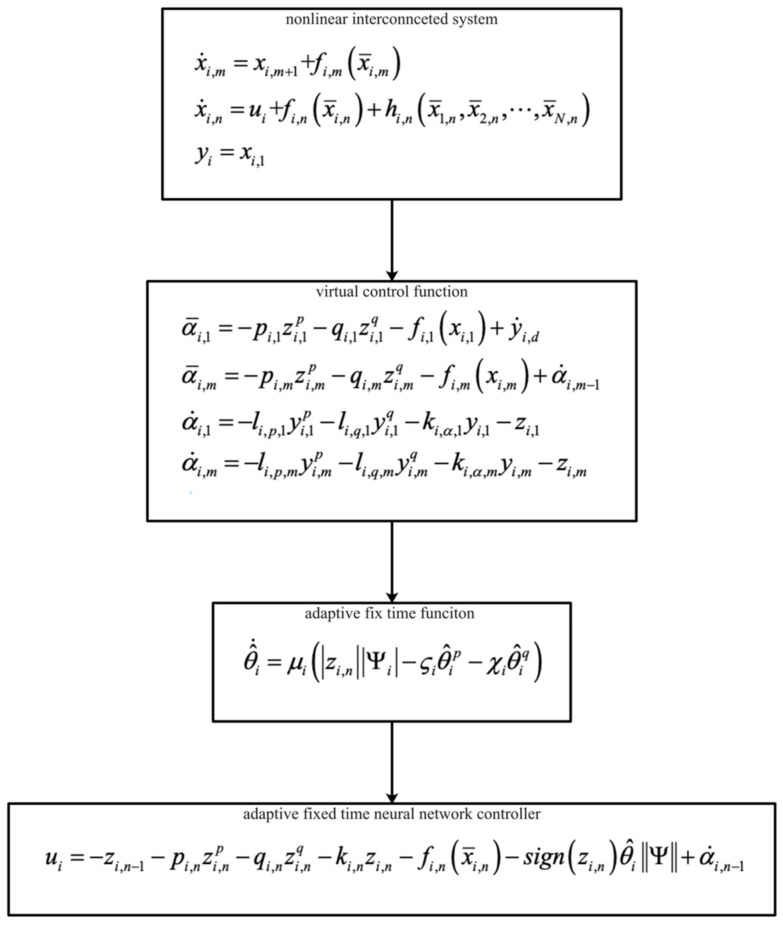
Design procedure.

**Remark** **4:** *The design details are summarized in Figure 2. The above-listed step procedure is detailed below.*

*Step 1: Design of the ideal virtual control laws (4) and (14), based on backstepping control technology.*

*Step 2: Design of the virtual control laws (5) and (15), based on the fixed-time low pass filter.*

*Step 3: The actual controller (25) is obtained recursively through the virtual control signal and the adaptive parameter (26).*

## 4. Numerical Examples

The main purposes of the simulation studies include: (1) validating the effectiveness of the adaptive fixed-time neural network tracking controller for nonlinear interconnected systems; (2) showing a step-by-step procedure for building the adaptive fixed-time tracking control system.

A. Numerical Example

Consider the following nonlinear interconnected system:

(37) $$\left\{\begin{array}{l} {\dot{x}}_{1,1}=x_{1,2}\\ {\dot{x}}_{1,2}=\sin\left(x_{1,1}\right)+u_{1}+h_{1,2}\\ y_{1}=x_{1,1}\\ {\dot{x}}_{2,1}=x_{2,2}\\ {\dot{x}}_{2,2}=\sin\left(x_{2,1}\right)+u_{2}+h_{2,2}\\ y_{2}=x_{2,1} \end{array}\right.$$

where the function, $h_{1,2}=\sin\left(x_{2,1}\right)-\sin\left(x_{1,1}\right)$. $h_{2,2}=\sin\left(x_{1,1}\right)-\sin\left(x_{2,1}\right)$.

Step 1: Design of the fixed-time ideal virtual control law and fixed-time adaptive law of neural networks:

(38) $${\bar{\alpha }}_{i,1}=-2z_{i,1}^{\frac{1}{3}}-2z_{i,1}^{3}$$

(39) $${\dot{\hat{\theta }}}_{i}=0.01\left(\left|z_{i}\right|\left|\Psi_{i}\right|-0.1{\hat{\theta }}_{i}^{\frac{5}{3}}-0.1{\hat{\theta }}_{i}^{\frac{1}{3}}\right)$$

Step 2: Design of the fixed-time low pass filter:

(40) $${\dot{\alpha }}_{i,1}=-2y_{i,1}^{\frac{1}{3}}-2y_{i,1}^{3}-2y_{i,1}-z_{i,1},\alpha_{i,1}\left(0\right)=0$$

Step 3: The actual controller is obtained recursively through the virtual control signal and the adaptive parameter, and the control input is designed as

(41) $$u_{i}=-z_{i,1}-2z_{i,2}^{\frac{1}{3}}-2z_{i,2}^{3}-2z_{i,2}-sign\left(z_{i,2}\right){\hat{\theta }}_{i}\left\|\Psi_{i}\right\|+{\dot{\alpha }}_{i,1}$$

The initial condition is selected as $x_{11}\left(0\right)=-2,x_{21}\left(0\right)=2,{\hat{\theta }}_{1}\left(0\right)=0,{\hat{\theta }}_{2}\left(0\right)=0$ The neural network consists of seven nodes, the centers $c=\left[-3,-2,-1,0,1,2,3\right]$, and the widths b=1, respectively.

**Figure 3:**
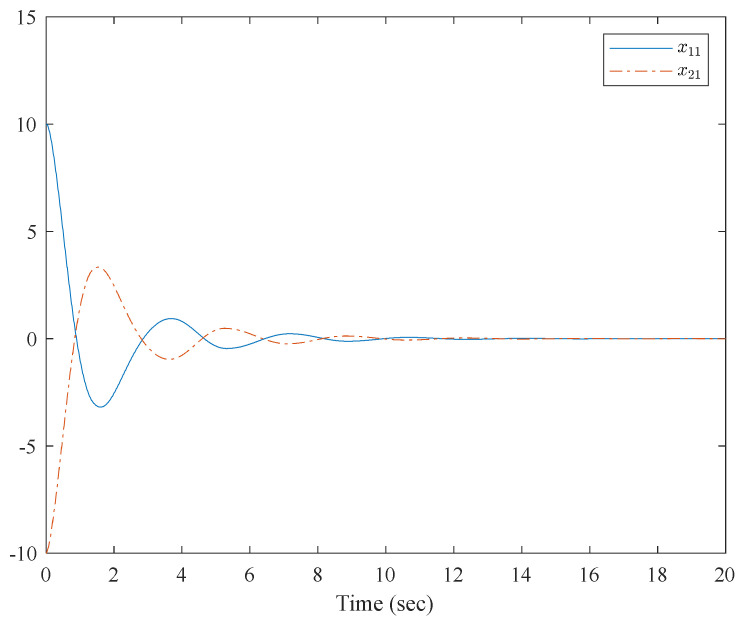
Trajectories of $x_{1,1}$ and $x_{2,1}$ of the interconnected system.

**Figure 4:**
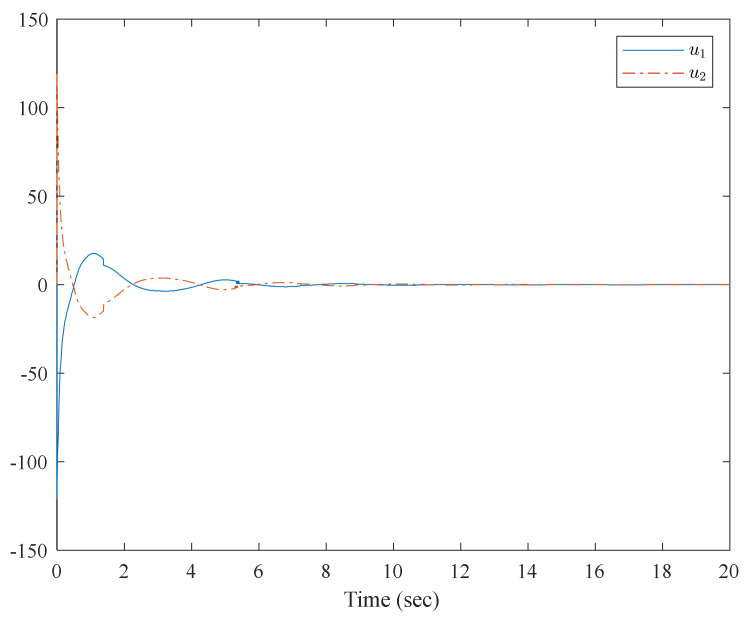
Trajectories of the controller of the interconnected system.

**Figure 5:**
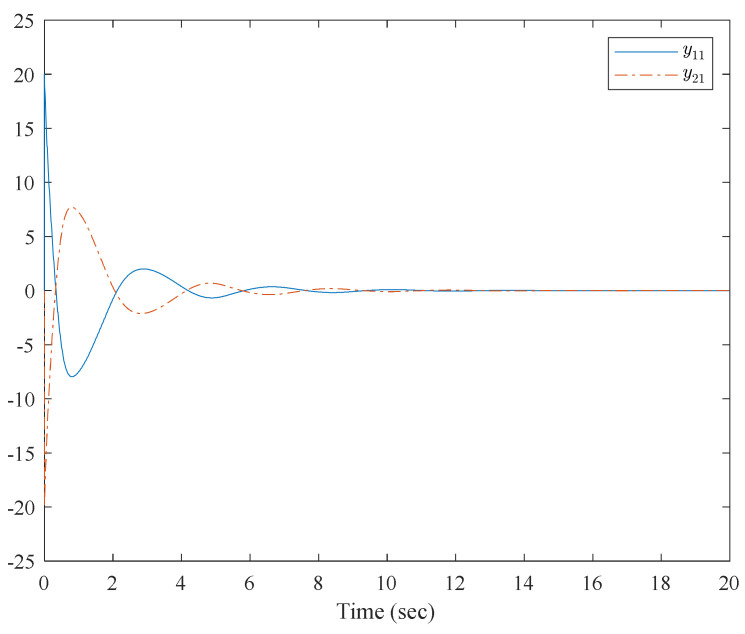
Trajectories of error of the low pass filter.

Figure 1 shows the control structure of the closed-loop system. Figure 2 shows the step-by-step design procedure. Figure 3, Figure 4 and Figure 5 are the simulation results. Figure 3 shows the output of the interconnected system and the output of the interconnected system’s convergence to the origin point in finite time, which indicates the control performance of the fixed-time neural network adaptive control. Figure 4 shows the controller of the interconnected system, which is bound and realizable. Figure 5 shows the trajectories of error of the low pass filter, and the error between the virtual control and ideal virtual control indicates that the virtual control is close to the ideal virtual control and that the differential coefficient exists. The simulation results show that the controlled system can become stable in 14 s, and the convergence time can be designed. Even if the states’ initial parameters change, the controlled system can still become stable in 14 s. The system includes unknown nonlinear functions, which verify that the neural network control scheme has strong adaptive ability and approximation ability.

B. Application Example

**Figure 6:**
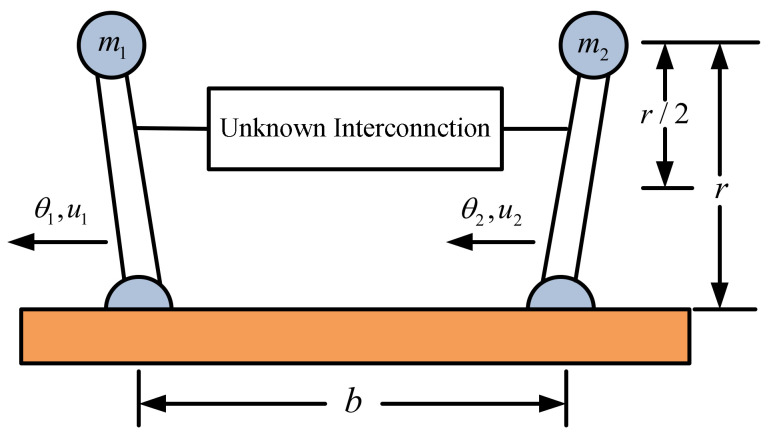
Two inverted pendulums connected by an unknown device.

Consider a pendulum system [25], with two degrees, as an interconnected system (Figure 6), which has been used as an example of decentralized neural control.

(42) $$\left\{\begin{array}{l} {\dot{x}}_{1,1}=x_{1,2}\\ {\dot{x}}_{1,2}=\frac{1}{J_{1}}u_{1}+\frac{kr}{2J_{1}}\left(l-b\right)+\left(\frac{m_{1}gr}{J_{1}}-\frac{kr^{2}}{4J_{1}}\right)\sin\left(x_{1,1}\right)+\frac{kr^{2}}{4J_{1}}\sin\left(x_{2,1}\right)\\ y_{1}=x_{1,1}\\ {\dot{x}}_{2,1}=x_{2,2}\\ {\dot{x}}_{2,2}=\frac{1}{J_{2}}u_{2}+\frac{kr}{2J_{2}}\left(l-b\right)+\left(\frac{m_{2}gr}{J_{2}}-\frac{kr^{2}}{4J_{2}}\right)\sin\left(x_{2,1}\right)+\frac{kr^{2}}{4J_{2}}\sin\left(x_{2,1}\right)\\ y_{2}=x_{2,1} \end{array}\right.$$

Step 1: Design of the fixed-time ideal virtual control law and fixed-time adaptive law of neural networks:

(43) $${\bar{\alpha }}_{i,1}=-2z_{i,1}^{\frac{1}{3}}-2z_{i,1}^{3}$$

(44) $${\dot{\hat{\theta }}}_{i}=0.01\left(\left|z_{i}\right|\left|\Psi_{i}\right|-0.1{\hat{\theta }}_{i}^{\frac{5}{3}}-0.1{\hat{\theta }}_{i}^{\frac{1}{3}}\right)$$

Step 2: Design of the fixed-time low pass filter:

(45) $${\dot{\alpha }}_{i,1}=-2y_{i,1}^{\frac{1}{3}}-2y_{i,1}^{3}-2y_{i,1}-z_{i,1},\alpha_{i,1}\left(0\right)=0$$

Step 3: The actual controller is obtained recursively through the virtual control signal and the adaptive parameter, and the control input is designed as

(46) $$u_{i}=-z_{i,1}-2z_{i,2}^{\frac{1}{3}}-2z_{i,2}^{3}-2z_{i,2}-sign\left(z_{i,2}\right){\hat{\theta }}_{i}\left\|\Psi_{i}\right\|+{\dot{\alpha }}_{i,1}$$

The initial condition is selected, and $m_{1}=2\;\mathrm{kg}$, $m_{2}=2.5\;\mathrm{kg}$, $J_{1}=0.5\;\mathrm{kg}$, $J_{2}=0.625\;\mathrm{kg}$, k=100 N/M, r=0.5 m, l=0.5 m, $g=9.81\;m/s^{2}$, b=0.4 m and the function, $y_{1,d}=0.5\sin\left(t\right)$. $y_{2,d}=0.5\cos\left(t\right)$ The neural network consists of seven nodes, the centers, $c=\left[-3,-2,-1,0,1,2,3\right]$ and the widths, respectively.

**Figure 7:**
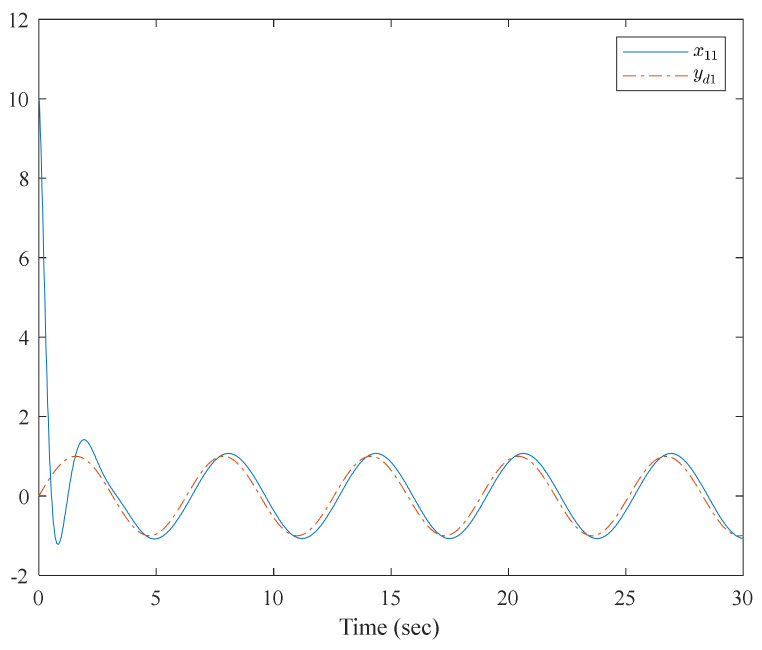
Trajectories of $x_{1,1}$ and $y_{1,d}$ of the interconnected system.

**Figure 8:**
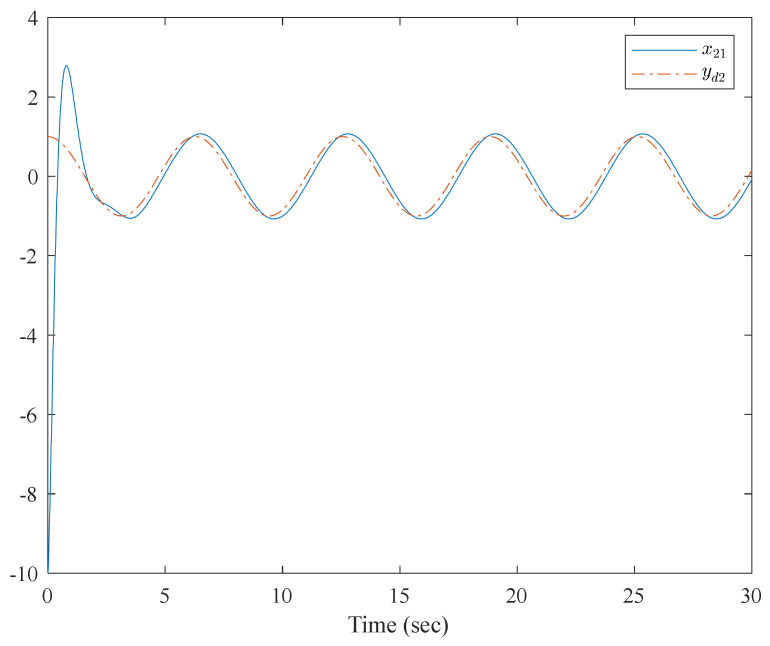
Trajectories of $x_{2,1}$ and $y_{2,d}$ of the interconnected system.

**Figure 9:**
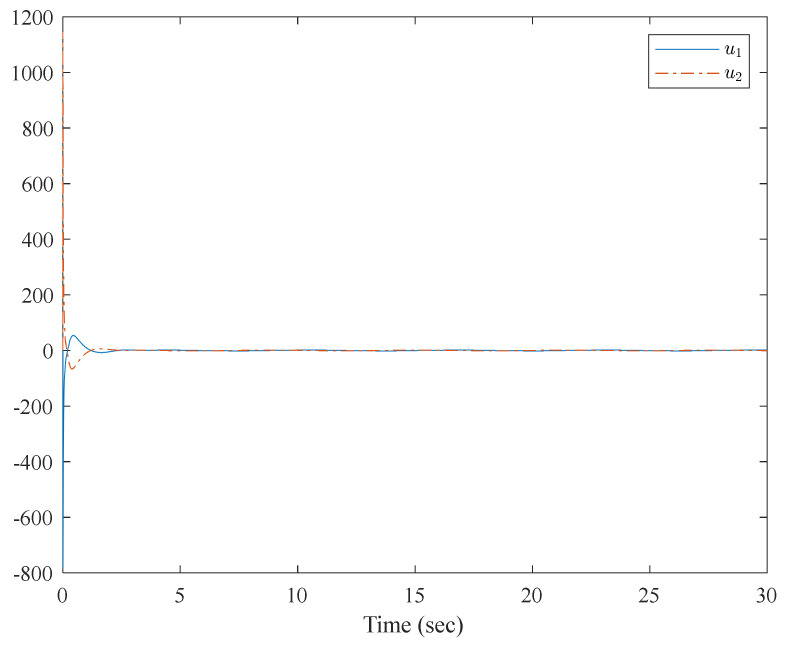
Trajectories of the controller of the interconnected system.

**Figure 10:**
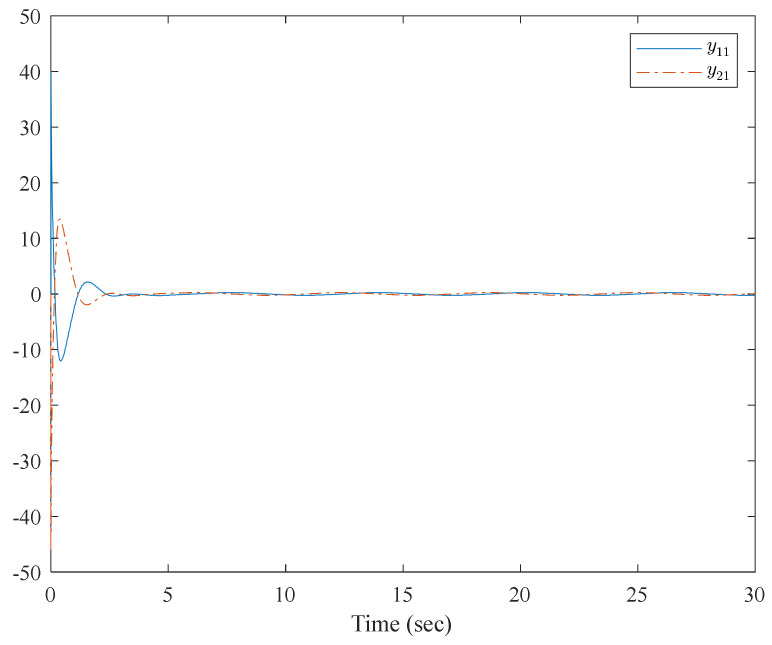
Trajectories of error of the low pass filter.

The simulation results are shown in Figure 7, Figure 8, Figure 9 and Figure 10. From the simulation results, it can be seen that the system output can track the desired signal in 2.5 and 5 s, respectively, and the setting time can be designed. Even if the states’ initial parameter change, the controlled system can still become stable in the same time. The pendulum system includes unknown nonlinear functions, which verify that the neural network control scheme has strong adaptive ability and approximation ability. The pendulum system also includes interconnected items, which verify that the control scheme can be applied to interconnected nonlinear control systems.

## 5. Conclusions

In this paper, through the design of a timing adaptive law for a neural network error system based on the Lyapunov fixed-time stability theorem, the unknown parameters of neural networks are iterated in fixed time. The convergence time can be designed only by modifying the parameters of the system controller and the adaptive rate, and it does not depend on the initial conditions. The fixed-time Lyapunov stability theorem is proposed, and the strict mathematical proof is completed, which will have more practical significance than the ideal stability analysis based on Lyapunov stability theory. Furthermore, the algorithm flow chart is given, which can be used by engineers to realize the proposed tracking control method by a computer for practical engineering. However, the control scheme based on neural networks also has some limitations. Firstly, because it cannot guarantee the asymptotic stability of the system, it is suitable for the controlled system, whose control objective is bounded stability. Secondly, the pure feedback structure is more general than strict feedback, and pure feedback has more application value; therefore, pure feedback interconnected system fixed-time neural network control results should be presented in future works.

## Data Availability

Not applicable.
